# Effects of the antihypertensive drug benidipine on osteoblast function *in vitro*

**DOI:** 10.3892/etm.2014.1475

**Published:** 2014-01-03

**Authors:** BAIXIANG WANG, MING BI, ZHEN ZHU, LEI WU, JINGYUN WANG

**Affiliations:** 1 Department of Prosthodontics, School of Stomatology, Jilin University, Changchun, Jilin 130021, P.R. China; 2 Department of Comprehensive Treatment, School of Stomatology, Jilin University, Changchun, Jilin 130021, P.R. China; 3 Department of Prosthodontics, Zhenjiang Stomatological Hospital, Zhenjiang, Jiangsu 212002, P.R. China

**Keywords:** benidipine, calcium channel blockers, osteoporosis, hypertension

## Abstract

The dihydropyridine-type calcium channel blocker, benidipine (BD) has been widely used in hypertension therapy. Previous studies have demonstrated that BD has a positive effect on bone metabolism. Inspired by this promoting phenomenon, the present study investigated the effects of BD on osteoblasts *in vitro*. Experiments were designed and performed, including an MTT assay, reverse transcription-polymerase chain reaction, western blot analysis, alkaline phosphatase activity measurements and alizarin red S staining. The results demonstrated that BD promoted osteoblast proliferation and osteogenic differentiation at concentrations from 1×10^−6^ to 1×10^−9^ M by upregulating Runx2, BMP2 and OCN gene expression levels. Overall, BD at appropriate concentrations has been demonstrated to have positive effects on osteoblast function in addition to its conventional clinical usage.

## Introduction

As one of the most common types of chronic disorder in aged people, osteoporosis has a multifactorial etiology and has been characterized by progressive bone substance loss, microarchitecture impairments and an increased risk of fractures (1,2). In addition to the systemic symptoms, patients with osteoporosis also suffer from the dental diseases periodontitis and dentition defect, which usually cause bone mass insufficiency (3,4). Due to impaired alveolar bone structure and metabolic disturbances, it is accordingly difficult to treat these patients. A great deal of effort has been made to alleviate this situation, yet few treatment technologies are applied widely. In current clinical practice, most dentists prefer to select drugs as facilitators (5). Osteoblasts usually originate from mesenchymal stem cells and are of importance during the bone formation process (6). Osteoblasts often behave abnormally in bone metabolism disorders. Therefore, numerous drugs for osteoporosis treatment are targeted at regulating osteoblast function.

Osteoblasts express several types of calcium channels. Of these channels, the L-type voltage-sensitive channel is the one most clearly involved in functional osteoblast regulation. A previous study has demonstrated that calcium channels are associated with proliferation, apoptosis and differentiation in osteoblasts (7). As a large number of patients who suffer from bone metabolism disorders also require hypertension treatment, the identification an appropriate antihypertensive drug that is able to also treat bone disorders would be of great significance. If a drug stimulates osteoblast function while performing an antihypertensive effect, it is likely to present a great benefit for elderly patients and doctors.

Benidipine (BD) is a dihydropyridine-type calcium channel blocker and has been widely used for hypertension therapy. It blocks the L-type and T-type calcium channels in different types of cells, including osteoblasts (8). Due to the dual effects of BD on hypertension and calcium channels, it is hypothesized to be a suitable candidate for the treatment of patients with osteoporosis and hypertension. Therefore, the aim of the present study was to evaluate the effect of BD at different concentrations on osteoblasts *in vitro*.

## Materials and methods

### Medicine preparation

A solution of BD (Kyowa Hakko Kirin Co., Ltd., Tokyo, Japan) was prepared by dissolving solid BD in dimethylsulfoxide (DMSO) solvent. The stock solution was stored at −20°C.

### Cell culture

MC3T3-E1 cells (American Type Culture Collection, Manassas, VA, USA) were cultured in α-MEM containing 100 U/ml penicillin, 100 U/ml streptomycin and 10% fetal bovine serum (FBS) in a humidified incubator at 37°C and 5% CO_2_. The cells were subcultured every three days in the presence of 0.25% trypsin.

### MTT assay

MC3T3-E1 cells were seeded in 96-well plates (5,000 cells/well) and incubated overnight. BD solution at different concentrations (final concentrations of 1×10^−4^–1×10^−10^ M) was then added. Cells without BD treatment were used as a negative control and wells without cells were set as blanks. One-, two-, and three-day further incubations were performed, and then 20 μl MTT (5.0 mg/ml) was added and the cells were incubated for another 4 h at 37°C. Subsequently, the supernatant was removed, DMSO was added and the optical density (OD) at 570 nm was measured on a microplate spectrophotometer (Model 680 Microplate Reader; Bio-Rad, Hercules, CA, USA). The proliferation rate of the cells was calculated according to the following formula: (OD_sample_ ‐ OD_blank_) / (OD_control_ ‐ OD_blank_).

### Assay for alkaline phosphatase (ALP) activity

MC3T3-E1 cells were seeded in 24-well plates (2×10^4^ cells/well) containing α-MEM medium and 10% FBS. After 24 h, the culture medium was changed to α-MEM, 10% FBS and osteogenic induction supplement containing 10 mmol/l disodium β-glycerophosphate and 0.15 mmol/l ascorbic acid (Sigma, St. Louis, MO, USA). A series of dilutions of BD (final concentrations, 1×10^−6^–1×10^−9^ M) were added to the culture medium in the 24-well plates for 3, 5, 7, 10 and 14 days. MC3T3-E1 cells treated with only osteogenic induction supplement were used as the control group. Following incubation, the MC3T3-E1 cells were washed twice with ice-cold PBS and lysed by two cycles of freezing and thawing. Aliquots of the supernatants were subjected to ALP activity and protein content measurement using an ALP activity kit and a bicinchonininc acid (BCA) protein assay kit (Nanjing Jiancheng Bioengineering Institute, Nanjing, China). All the results were normalized by protein content.

### Assay for mineralized matrix formation

Cells were seeded in 24-well plates (2×10^4^ cells/well) and cultured overnight at 37°C in a 5% CO_2_ humidified incubator. The medium was then changed to medium containing osteogenic induction supplement and BD (1×10^−6^–1×10^−9^ M) for 21 days. The formation of mineralized matrix nodules was determined by alizarin red S (ARS) staining. Briefly, the cells were fixed in 95% ethanol for 30 min at room temperature. The fixed cells were washed with PBS and stained with 1% ARS (pH 4.2) for 30 min at room temperature. Quantitative analysis was performed by elution with 10% (w/v) cetylpyridium chloride for 10 min at room temperature, and the OD was measured at 570 nm.

### RNA isolation and semiquantitative reverse transcription-polymerase chain reaction (RT-PCR)

Total RNA was extracted following three-day incubation using TRIzol reagent (Invitrogen Life Technologies, Carlsbad, CA, USA). Complementary DNA (cDNA) was produced using a transcriptase PCR kit (ReverTra Dash; Toyobo Biochemicals, Osaka, Japan). Aliquots of total cDNA were amplified, using PCR equipment (PC701 thermal cycler; Astec, Fukuoka, Japan). The amplification reaction products were resolved on 1.5% agarose/TAE gels by electrophoresis at 100 mV, and were visualized by ethidium-bromide staining. The primers used are presented in Table I.

### Western blot analysis

MC3T3-E1 cells were washed with cold PBS and lysed in cold Tris-HCl (50 mM, pH 7.4), 10 mM EDTA, 4.3 M urea and 1% Triton X-100. Proteins were subjected to SDS-PAGE using 10% separation gel and transferred to a nitrocellulose membrane. The membrane was blocked for 2 h at room temperature with 5% bovine serum albumin in TBST solution (10 mM Tris-HCl, pH 8.0; 150 mM NaCl; 0.05% Tween-20). Subsequently, the blots were incubated with the corresponding primary antibodies (rabbit anti-Runx2, rabbit anti-BMP2 and rabbit anti-OCN) (Biosynthesis Biotechnology Co., Ltd., Beijing, China) in the TBST solution overnight at 4°C, followed by 2 h incubation with secondary goat anti-rabbit IgG antibodies (Santa Cruz Biotechnology Inc., Santa Cruz, CA, USA) conjugated with horseradish peroxidase, and visualized with an enhanced luminol-based chemiluminescent (ECL) kit (Thermo Fisher Scientific Inc., Waltham, MA, USA). The OD of the bands was quantified using LAS-1000 luminescent image analyzer software (Fujifilm, Berlin, Germany).

### Statistical analysis

One-way analysis of variance and Tukey’s multiple comparison tests were performed to detect any significant effects that occurred as a result of the experimental variables. All results are expressed as the mean ± standard deviation. P<0.05 was considered to indicate a statistically significant difference.

## Results

### Effect of BD on the proliferation of MC3T3-E1 cells

**Figure 1:**
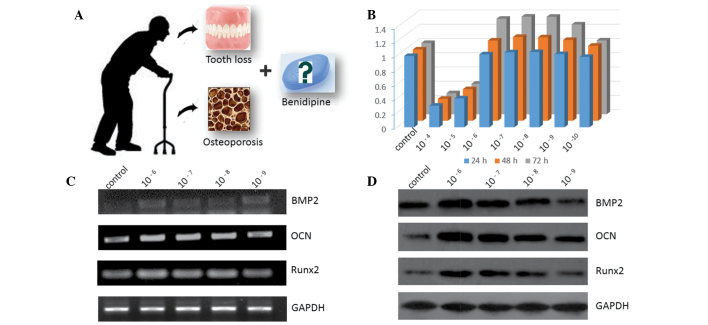
(A) Schematic diagram demonstrating that patients with osteoporosis suffer from systemic and dental symptoms. (B) The osteoblast proliferation rate is increased in the presence of BD at certain concentrations over a three day period. Inhibition of proliferation was observed when higher concentrations of BD were applied to the cells. Data are presented as the mean ± SD. (C) Gene and (D) protein expression levels of BMP2, OCN and Runx2 were upregulated when MC3T3-E1 cells were treated with BD for three days. BD, Benidipine.

As demonstrated in Fig. 1B, the effect of BD on the proliferation of MC3T3-E1 cells was time-dependent and the proliferation rate decreased with increasing BD concentrations. Following one-, two- and three-day treatment, BD promoted proliferation at concentrations of 1×10^−6^–1×10^−9^ M. The higher concentrations of BD inhibited cell proliferation whereas no significant difference from the control was observed when the lower concentrations of BD were applied.

### Effect of BD on gene and protein expression

Following treatment with different concentrations of BD for three days, BMP2, OCN and Runx2 mRNA levels were markedly upregulated compared with those in the control group in a concentration-dependent manner (Fig. 1C). As demonstrated in Fig. 1D, BMP2, OCN and Runx2 protein levels were enhanced following BD treatment; the most prominent enhancements were observed in the groups of cells treated with 1×10^−6^ and 1×10^−7^ M BD.

### Effect of BD on the differentiation of MC3T3-E1 cells

**Figure 2:**
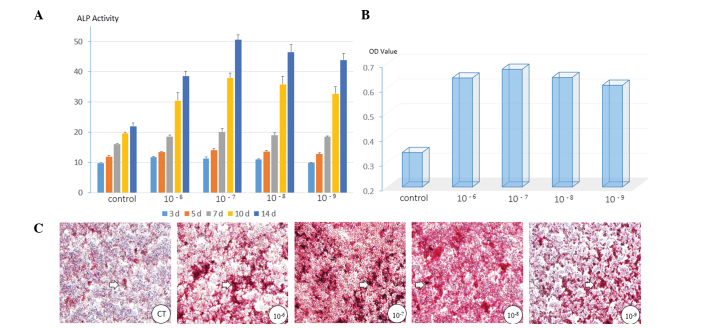
(A) ALP activity was enhanced significantly in a time-dependent manner when osteoblasts were treated with BD. (B) Mineralization nodules were dissolved by cetylpyridium chloride and the OD value was measured at 570 nm. The results demonstrated prominent enhancement of mineralization when BD was applied. (C) ARS staining was conducted following 21-day osteogenic induction. The white arrows indicate the mineralization nodules. Data are presented as the mean ± SD. ALP, alkaline phosphatase; OD, optical density; CT, control; BD, Benidipine; ARS, Alazarin red S.

As presented in Fig. 2A, all four groups treated with BD exhibited elevated levels of ALP activity compared with those in the control group. The highest level was observed in the cells treated with 1×10^−7^ M BD. From day 3 to day 14, the level of ALP activity in each of the groups of cells increased in a time-dependent manner.

BD also promoted the formation of mineralized matrix nodules in the MC3T3-E1 cells. The results of the quantitative analysis of the ARS staining were in accordance with the morphological observations. BD at 1×10^−7^ M also resulted in the clearest promotive effect in the cells (Fig. 2B and C).

## Discussion

Cell proliferation is a key attribute of the bone repair process. In the present study, it was demonstrated that the effects of BD on the proliferation of MC3T3-E1 cells varied according to the concentration of BD. A low concentration of BD had no effect on cell proliferation. At a BD concentration of 1×10^−6^ M, cell proliferation was promoted and this effect was observed in the cells treated with concentrations down to 1×10^−9^ M BD, whereas inhibition was observed when cells were treated with the higher concentrations. Thus, the concentrations 1×10^−6^–1×10^−9^ M BD were selected for the remaining experiments. The result is inconsistent with a previous study (9) and this may be attributable to the different techniques applied. The inhibitory effect is likely to be the result of cytotoxicity.

A large number of proteins that have been associated with bone cells are specifically required for osteoblast differentiation, such as Runx2, BMP2 and OCN (10). Runx2 is a master regulator of osteogenic gene expression and osteoblast differentiation. It has been reported that Runx2 knockout mice exhibit no bone tissues or osteoblasts, indicating that osteoblast differentiation is completely blocked in the absence of Runx2 (11,12). In addition to being required for osteoblast differentiation, Runx2 is necessary for the proper function of mature osteoblasts, including the synthesis of bone matrix (13).

OCN is the most specific gene for osteoblast differentiation and mineralization. OCN is expressed during the postproliferative period, reaches its maximum expression during mineralization and accumulates in the mineralized bone (14). BMP2 is a member of the transforming growth factor-β superfamily and has a key regulatory role as a cell-cell signaling molecule during bone formation and repair. BMP2, which is a potent osteogenic protein required for osteoblast differentiation and bone formation, induces low levels of expression of osteoblast marker genes such as OCN and ALP in calvarial cells from Cbfa1^−/−^ animals (12). Furthermore, it has been shown that mice lacking BMP2 in the limb mesenchyme exhibit a clear defect in bone mineral density shortly after birth, indicating that BMP2 has a unique role in bone formation (15).

ALP, a cell membrane-associated enzyme, appears early during osteoblast differentiation and is the most widely recognized marker of osteoblastic differentiation (16). ALP activity correlates with matrix formation in osteoblasts prior to the initiation of mineralization. In the present study, BD enhanced ALP activity at five time points in a time-dependent manner, but no significant concentration-dependent manner was observed. While the appearance of ALP activity is an early marker of differentiation, mineralized nodule formation is considered as a late marker for maturation (17,18). Consistent with the ALP activity result, an increased mineralization level was observed in the BD-treated cells. This was further confirmed by the quantitative analysis.

In addition to the conventional antihypertensive function of BD, several studies have suggested that BD increases the ALP activity of osteoblastic cells and also stimulates mineral matrix deposition (19,20). In addition, BD has been shown to decrease receptor activator of nuclear factor κB ligand expression in human osteoblasts, indicating the suppression of osteoclast differentiation (21). The systematic experiments conducted in the present study also demonstrated that BD promoted the proliferation, osteogenic differentiation and mineralization of MC3T3-E1 cells at the cellular and molecular levels, when applied at concentrations of 1×10^−6^–1×10^−9^ M. These findings indicate that BD may be a novel candidate for the combined treatment of osteoporosis and hypertension. BD promoted osteogenesis most markedly at concentrations of 1×10^−7^ and 1×10^−8^ M, which is in accordance with the serum drug levels for antihypertensive therapy (22).

The results of the present study demonstrated that BD promotes cell proliferation and osteogenic differentiation at concentrations from 1×10^−6^ to 1×10^−9^ M by upregulating Runx2, BMP2 and OCN gene expression levels. Therefore, it was concluded that BD at the appropriate concentrations may have a positive effect on osteoblast function in addition to its conventional usage, and may be a suitable candidate for the treatment of patients with osteoporosis and hypertension.

## Figures and Tables

**Table I:** Primer sequences used for RT-PCR.

Gene	Forward (5′-3′)	Reverse (5′-3′)
Runx2	TTCTCCAACCCACGAATGCAC	CAGGTACGTGTGGTAGTGAGT
BMP2	TGGCCCATTTAGAGGAGAACC	AGGCATGATAGCCCGGAGG
OCN	GAACAGACTCCGGCGCTA	AGGGAGGATCAAGTCCCG
GAPDH	GACTTCAACAGCAACTCCCAC	TCCACCACCCTGT TGCTGTA

RT-PCR, reverse transcription-polymerase chain reaction.
